# A Missense *LRRK2* Variant Is a Risk Factor for Excessive Inflammatory Responses in Leprosy

**DOI:** 10.1371/journal.pntd.0004412

**Published:** 2016-02-04

**Authors:** Vinicius M. Fava, Jérémy Manry, Aurélie Cobat, Marianna Orlova, Nguyen Van Thuc, Nguyen Ngoc Ba, Vu Hong Thai, Laurent Abel, Alexandre Alcaïs, Erwin Schurr, Canadian Lrrk2 in Inflammation Team (CLINT)

**Affiliations:** 1 Program in Infectious Diseases and Immunity in Global Health, Research Institute of the McGill University Health Centre, Montreal, Canada; 2 The McGill International TB Centre, Departments of Human Genetics and Medicine, McGill University, Montreal, Canada; 3 Laboratory of Human Genetics of Infectious Diseases, Necker Branch, Institut National de la Santé et de la Recherche Médicale U1163, Paris, France; 4 University Paris Descartes, Imagine Institute, Paris, France; 5 Hospital for Dermato-Venerology, Ho Chi Minh City, Vietnam; 6 St. Giles Laboratory of Human Genetics of Infectious Diseases, Rockefeller Branch, Rockefeller University, New York, New York, United States of America; 7 Centre d’Investigation Clinique, Unité de Recherche Clinique, Necker and Cochin Hospitals, Paris, France; Fondation Raoul Follereau, FRANCE

## Abstract

**Background:**

Depending on the epidemiological setting, a variable proportion of leprosy patients will suffer from excessive pro-inflammatory responses, termed type-1 reactions (T1R). The *LRRK2* gene encodes a multi-functional protein that has been shown to modulate pro-inflammatory responses. Variants near the *LRRK2* gene have been associated with leprosy in some but not in other studies. We hypothesized that *LRRK2* was a T1R susceptibility gene and that inconsistent association results might reflect different proportions of patients with T1R in the different sample settings. Hence, we evaluated the association of *LRRK2* variants with T1R susceptibility.

**Methodology:**

An association scan of the *LRRK2* locus was performed using 156 single-nucleotide polymorphisms (SNPs). Evidence of association was evaluated in two family-based samples: A set of T1R-affected and a second set of T1R-free families. Only SNPs significant for T1R-affected families with significant evidence of heterogeneity relative to T1R-free families were considered T1R-specific. An expression quantitative trait locus (eQTL) analysis was applied to evaluate the impact of T1R-specific SNPs on *LRRK2* gene transcriptional levels.

**Principal Findings:**

A total of 18 T1R-specific variants organized in four bins were detected. The core SNP capturing the T1R association was the *LRRK2* missense variant M2397T (rs3761863) that affects LRRK2 protein turnover. Additionally, a bin of nine SNPs associated with T1R were eQTLs for *LRRK2* in unstimulated whole blood cells but not after exposure to *Mycobacterium leprae* antigen.

**Significance:**

The results support a preferential association of *LRRK2* variants with T1R. LRRK2 involvement in T1R is likely due to a pathological pro-inflammatory loop modulated by LRRK2 availability. Interestingly, the M2397T variant was reported in association with Crohn’s disease with the same risk allele as in T1R suggesting common inflammatory mechanism in these two distinct diseases.

## Author Summary

A major challenge of current leprosy control is the management of host pathological immune responses coined Type-1 Reactions (T1R). T1R are characterized by acute inflammatory episodes whereby cellular immune responses are directed against host peripheral nerve cells. T1R affects up half of all leprosy patients and are a major cause of leprosy-associated disabilities. Since there is evidence that host genetic factors predispose leprosy patients to T1R, we have conducted a candidate gene study to test if *LRRK2* gene variants are T1R risk factors. The choice of LRRK2 was motivated by the fact that *LRRK2* was associated with leprosy *per se* in some but not in other studies. We reasoned that this may reflect different proportions of leprosy patients with T1R in the different samples and that *LRRK2* may in truth be a T1R susceptibility gene. Here, we show that variants overlapping the *LRRK2* gene, reported as suggestive leprosy *per se* susceptibility factors in a previous genome-wide association study, are preferentially associated with T1R. The main SNP carrying most of the association signal is the amino-acid change M2397T (rs3761863) which is known to impact LRRK2 turnover. Interestingly, eQTL SNPs counterbalanced the effect of the M2397T variant but this compensatory mechanism was abrogated by *Mycobacterium leprae* antigen stimulation.

## Introduction

Leprosy is a chronic dermato-neurological infectious disease caused by *M*. *leprae*. Leprosy irrespective of its clinical presentation is curable by multi-drug therapy. The current global effort in leprosy control is focused on early recognition of leprosy cases and prevention of permanent disabilities [1]. A common complication of leprosy are excessive pro-inflammatory episodes termed type-1 reactions (T1R) [2]. If untreated, T1R can lead to irreversible nerve function impairment due to a pathological cellular immune response directed against host peripheral nerve cells [3]. Up to 50% of all leprosy cases can undergo T1R with the incidence varying according to endemic settings and criteria for case definition [4].

Why only a proportion of leprosy patients undergo T1R is not known. However, clinical and environmental factors have been associated with T1R outcome [5, 6]. Individuals categorized as borderline in the Ridley and Jopling clinical spectrum of leprosy are at increased risk to develop T1R while patients with tuberculoid or lepromatous polar leprosy forms rarely develop T1R [6]. Positive bacillary index, PCR detection of *M*. *leprae*, and increased age at leprosy diagnosis are other factors associated with T1R-risk [6–8]. A number of studies have shown a consistent upregulation of pro-inflammatory cytokines, i.e. TNF, IFNγ and the chemokine IP10, in the blood of T1R patients [4, 9–12]. In a prospective study, the transcriptional profile of leprosy cases destined for T1R displayed a distinct signature from leprosy patients that remained T1R-free [11]. A dysregulated balance between innate pro- and anti-inflammatory responses emerged from this study as a key factor in T1R outcome [11].

A number of genes have been shown to be associated with T1Rincluding *TNFSF15* and the pathogen recognition genes *TLR1*, *TLR2* and *NOD2* [3, 13]. All of these genes had also been found to be associated with leprosy *per se* in other studies [14–16]. Since leprosy patients are usually not stratified by their T1R status, it is possible that some of the leprosy *per se* associations were caused by T1R patient subgroups. For example, the *TNFSF15* gene had initially been shown to be associated with leprosy *per se* by a genome wide association study (GWAS) in a Chinese population [14]. However, in a Vietnamese sample stratified by T1R status the association signal could be unambiguously assigned to the T1R group [13]. Of the genes reported by the GWAS, the *TNFSF15* and *LRRK2* were the only leprosy susceptibility genes not validated for association with leprosy *per se* in a Vietnamese population [17]. Like *TNFSF15*, *LRRK2* is a gene with an uncertain role in leprosy *per se* susceptibility. Several groups have evaluated the association of the *LRRK2* gene with leprosy susceptibility but results were inconsistent [17–20]. Given that T1R affects different proportions of leprosy cases according to the studied population, we wondered if inconsistencies in *LRRK2* association with leprosy *per se* were due to different proportions of T1R in each setting. Here, we evaluated a possible role for *LRRK2* in T1R-affected families and contrasted the results with T1R-free families. We identified a set of 18 SNPs in *LRRK2* preferentially associated with T1R. These variants overlapped with previous associations reported for Crohn’s Disease (CD), Ulcerative Colitis (UC) and Inflammatory Bowel Disease (IBD) [21, 22].

## Methods

### Population sample

**Fig 1:**
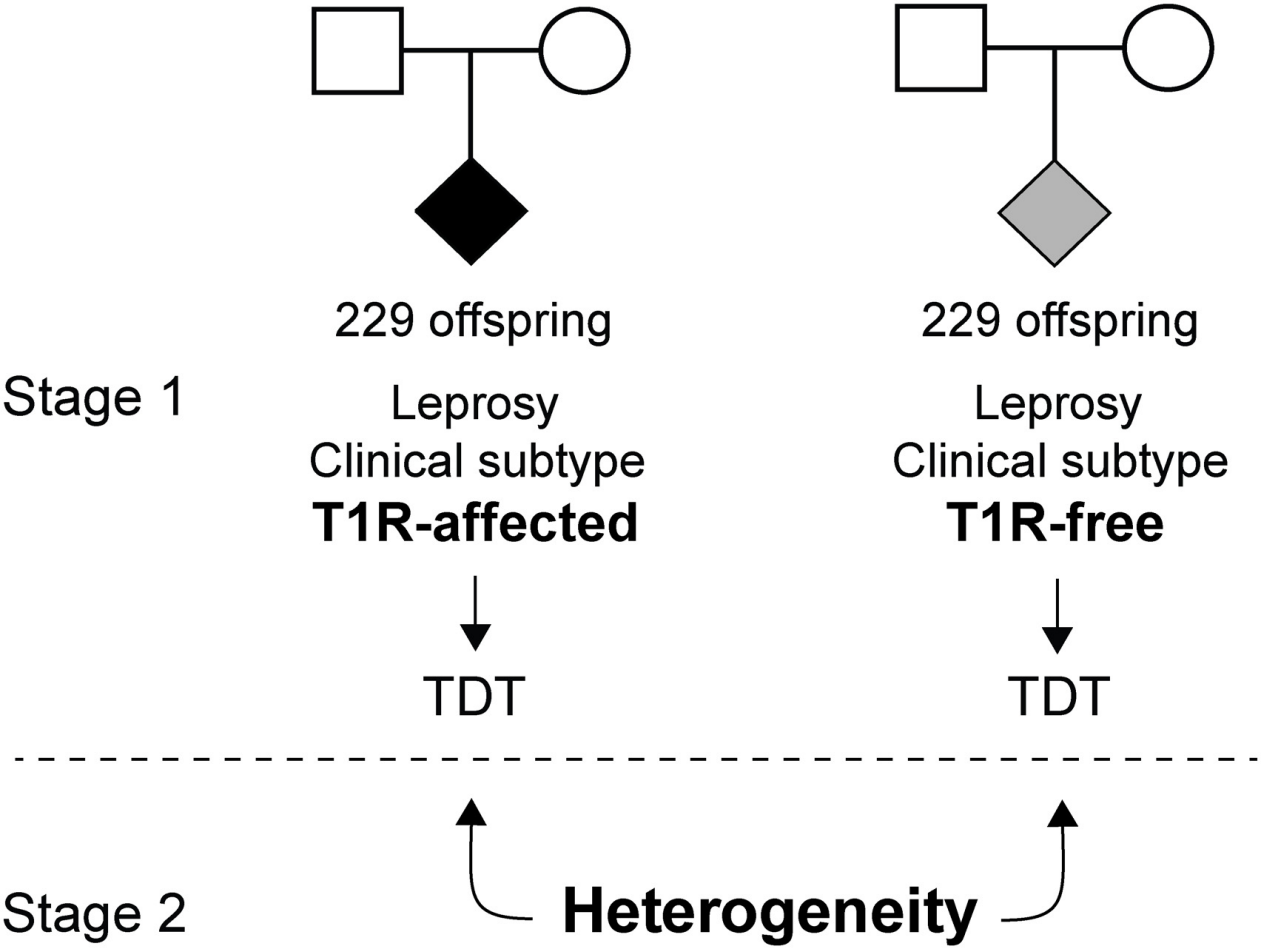
Family based sample and study design. Two sets of families were employed: those with T1R-affected offspring and those with leprosy but T1R-free offspring. The T1R-affected subset comprised 229 offspring belonging to 221 families while the T1R-free subset comprised 229 offspring in 209 families. Offspring were matched by clinical leprosy subtype in the two family sets. In a first analysis stage, the transmission disequilibrium test (TDT) was used to estimate significance of association of *LRRK2* variants with disease in each subset. In a second stage, a formal heterogeneity test was performed to identify *LRRK2* variants preferentially associated with T1R.

For the *LRRK2* study, a total of 1372 individuals were selected [13]. These individuals were divided in two family-based samples. The first set of families contained 229 leprosy affected offspring that underwent T1R (T1R-affected) and their respective parents. The T1R-free subset was matched to the T1R-affected subset by leprosy clinical subtype of the offspring (Fig 1). Consequently, the second set of families included 229 leprosy affected offspring and their parents in which the offspring had no signs of leprosy reaction (T1R-free). There was no difference in gender and age at leprosy onset regarding T1R outcome between both subsets (S1 Table). The subjects included in the eQTL analyses were part of a study evaluating the transcriptional profile of leprosy patients prior to T1R onset [23]. Briefly, 53 newly diagnosed leprosy cases in the borderline spectrum (19 BT, 30 BB and 4 BL) were enrolled. A blood sample was collected from each subject within 3 months of leprosy diagnosis and none of the subjects suffered T1R at enrolment.

### Ethics statement

The samples used in the current study were selected from our records at the Dermato Venerology Hospital, Ho Chi Ming City, Vietnam, as described previously [13]. Written informed consent was obtained from all subjects enrolled in the study and all subjects were anonymized. This study was approved by the regulatory authorities in Ho Chi Minh City, Vietnam, and the Research Ethics Board at the Research Institute of the McGill University Health Centre, Montreal, Canada. The investigation have been conducted according to the principles expressed in the Declaration of Helsinki.

### Genotyping and expression data

Genotypes for156 SNPs mapping to a 500 kb window overlapping the *LRRK2* and *MUC19* genes were obtained via the 660W-quad v1 Illumina array [24]. The variants selected covered 89% of the SNPs with a MAF > 5% at a *r*^2^ > 0.5 for the Vietnamese (KHV) and Chinese (CHB) and 84% for Caucasians (CEU) populations from the 1000 genomes project [25]. All genotypes passed standard quality-control presenting call rates greater than 98%, less than 2 Mendelian Errors (ME) and were in Hardy-Weinberg Equilibrium (HWE) with *P* > 0.05 in 763 leprosy unaffected parents from both T1R-affecte and T1R-free subsets. *LRRK2* expression levels were obtained using Illumina HumanHT12 v4 BeadChips as previously described [23]. Briefly, whole blood from the 53 leprosy patients was divided in two aliquots. One aliquot was stimulated with *M*. *leprae* sonicate for 24hrs to 30 hrs while the second aliquot was incubated for the same time interval in the absence of *M*. *leprae* sonicate (non-stimulated). Total RNA was extracted from all aliquots and used for *LRRK2* quantification.

### Study design and statistical approach

Family based SNP and haplotype association tests were performed using Transmission Disequilibrium Test (TDT) as implemented in FBAT 2.0.4 [26]. Association testing was carried out under the same genetic model in T1R-affected and T1R-free families and the *P* values for the best genetic model (additive or dominant) were displayed (Fig 1). Due to the highly correlated nature of the genotyped SNPs, we did not perform a correction for multiple testing. Subsequently, a formal heterogeneity test was performed to evaluate preferential association of genetic variants with T1R (Fig 1) by using a modified version of the FBAT statistics (FBAT_Het_) as described by Gaschignard, J. et al [27]. A multivariate analysis was performed to test for independence of T1R-specific associations. For each SNP bin (*r*^2^ > 0.5), SNP with the most significant evidence for association was included in the multivariate model. Multivariate analyses were done by stepwise conditional logistic regression (SAS v.9.3). Logistic regression was also used to estimate the odds ratio for each individual SNP in the T1R-affected subset. Briefly, the TDT evaluates the non-random transmission of alleles from heterozygote parents to affected offspring. We used the non-transmitted alleles from the TDT to create up to three unaffected pseudo-sibs per family, one for each possible genotype. We compared the original T1R-affected offspring with T1R-unaffected pseudo sibs in a matched case-control design as described in [28]. Under the additive model, the TDT and the conditional logistic regression statistics result in the same *P* values. The recombination rate in centimorgan by mega base (cM/Mb) according to the 1000 genomes and the LD structure of the *LRRK2/MUC19* locus were obtained with the R packages Locuszoom v.1.3 [29] and snp.ploter v.0.5.1 [30], respectively. The minor allele frequencies (MAF) and HWE for each SNP were estimated using Haploview 4.2 [31]. For the eQTL analyses, the correlation between genotypes and gene expression levels was performed with a simple linear regression under both stimulated and non-stimulated conditions using R 3.2.0.

### Parkinson’s disease and inflammatory bowel disease data

Genotype associations of *LRRK2* and PD were obtained from the PDgene database (www.pdgene.org). *LRRK2* variants associated with PD were obtained from a GWAS meta-analysis of 14 studies with a total population sample of 12,771 PD cases and 93,386 controls [32]. Genotype associations of *LRRK2* variants with IBD and CD were obtained from the IBDgenetics database (www.ibdgenetics.org). The IBDgenetics population sample consisted of 42,950 IBD cases (22,575 CD and 20,417 UC patients) and 53,536 controls [21, 22].

## Results

### *LRRK2* variants described in association with leprosy *per se* are associated with T1R

A genome-wide association (GWAS) in a Chinese sample provided the most comprehensive study of *LRRK2* in leprosy. Hence, we first analyzed six *LRRK2* SNPs (rs1873613, rs10878220, rs1491938, rs12820920, rs11174812 and rs11173979) described in association with leprosy *per se* by a GWAS in a Chinese population. None of the six variants presented significant evidence of association with leprosy in the T1R-free subset (Table 1). In contrast, SNPs rs10878220, rs1491938 and rs12820920 that belonged to the same r^2^ < 0.5 SNP bin were nominally associated with disease in the T1R-affected subset (Table 1). These SNPs were preferentially associated with T1R when a formal heterogeneity test was performed (Table 1). Moreover, the direction of association was the same as previously reported for leprosy *per se*. While SNP rs10878220 is located in the core promoter region of the *LRRK2* gene, SNPs rs1491938 and rs12820920 are *LRRK2* intronic variants (Table 1). The remaining three SNPs that did not show association with T1R are located 66kb to 367kp upstream of *LRRK2* transcription start site (Table 1).

**Table 1:** LRRK2 variants preferentially associated with T1R.

					T1R-affected			T1R-free			
SNP	Distance to *LRRK2* TSS (Kb)	*M/m*	LD bin (*r*^2^ > 0.5)	LD bin (*r*^2^ > 0.8)	RA (*f*)	OR (95% CI)	*P*	RA (*f*)	*P*	*P*_Het_	Feature
rs11173979	-367.5	C/T	-	-	T (0.15)	n.s.	0.51	T (0.15)	0.39	0.78	
rs11174812	-193.7	G/A	-	-	G (0.78)	n.s.	0.16	A (0.22)	0.61	0.29	-
rs7295598	-94.6	C/T	Bin 1	Bin 1	C (0.69)	2.29 (1.10–4.78)	0.01	T (0.31)	0.18	0.007	-
rs7311031	-93.2	T/C	Bin 1	Bin 1	T (0.70)	2.87 (1.27–6.49)	0.005	C (0.30)	0.26	0.005	-
rs1031996	-91.4	C/T	Bin 1	Bin 1	C (0.70)	2.87 (1.27–6.49)	0.005	T (0.30)	0.21	0.003	-
rs1873613	-66.4	A/G	-	-	A (0.68)	n.s.	0.13	G (0.32)	0.14	0.04	-
rs10878220	-9.9	C/T	Bin 2	Bin 5	C (0.67)	1.37 (1.02–1.83)	0.02	T (0.33)	0.52	0.004	eQTL
rs10878249	14.5	T/C	Bin 2	Bin 6	T (0.65)	1.40 (1.05–1.87)	0.01	C (0.35)	0.39	0.02	eQTL
rs2404580	20.7	T/C	Bin 2	Bin 6	T (0.65)	1.45 (1.08–1.95)	0.006	C (0.35)	0.71	0.01	eQTL
rs1491938	26.8	G/A	Bin 2	Bin 6	G (0.65)	1.42 (1.06–1.89)	0.007	A (0.35)	0.81	0.02	eQTL
rs10784470	44.8	G/T	Bin 2	Bin 6	G (0.65)	1.39 (1.04–1.86)	0.01	T (0.35)	0.39	0.02	eQTL
rs10506151	52.2	C/A	Bin 2	Bin 7	C (0.76)	1.57 (1.13–2.18)	0.004	C (0.76)	0.62	0.03	eQTL
rs11175847	57.4	G/T	Bin 2	Bin 6	G (0.65)	1.37 (1.02–1.84)	0.02	T (0.35)	0.67	0.03	eQTL
rs12820920	69.3	T/C	Bin 2	Bin 6	T (0.65)	1.37 (1.02–1.84)	0.02	C (0.35)	0.67	0.03	eQTL
rs4768230	96.5	G/A	Bin 2	Bin 6	G (0.65)	1.38 (1.03–1.85)	0.02	A (0.35)	0.43	0.02	eQTL
rs1427271	113.6	G/A	Bin 3	Bin 3	A (0.36)	1.70 (1.04–2.76)	0.01	G (0.64)	0.67	0.04	
rs10735934	114.1	G/T	Bin 2	Bin 2	G (0.54)	1.41 (1.06–1.87)	0.01	T (0.46)	0.67	0.04	
rs4768236	137.7	G/T	Bin 2	Bin 2	G (0.51)	1.49 (1.12–1.97)	0.003	T (0.49)	0.45	0.009	-
rs3761863	139.8	M/T	Bin 2	Bin 2	M (0.51)	1.49 (1.12–1.97)	0.003	T (0.49)	0.41	0.008	Protein turn-over
rs3886747	143.1	C/T	Bin 2	Bin 2	C (0.51)	1.49 (1.12–1.97)	0.003	T (0.49)	0.30	0.005	
rs1463739	340.4	A/G	Bin 4	Bin 4	A (0.74)	2.46 (1.08–5.62)	0.04	G (0.26)	0.11	0.01	

T1R, type-1 reaction; LRRK2, leucine-rich repeat kinase 2; TSS, transcription starting site; Kb, kilo-base; M, major allele; m, minor allele; LD, linkage disequilibrium; RA, risk-allele; f, allele frequency; OR, odds ratio; CI, confidence interval; ns, non-significant.

Linkage disequilibrium and allele frequencies were estimated in 763 leprosy unaffected parents; T1R-affected and T1R-free were compared under the same genetic model; P Het, P values for the formal heterogeneity test between T1R-affected and T1R-free subsets; a GWAS SNPs associated with leprosy per se in the Chinese population.

### Fine mapping the *LRRK2* association with T1R

**Fig 2:**
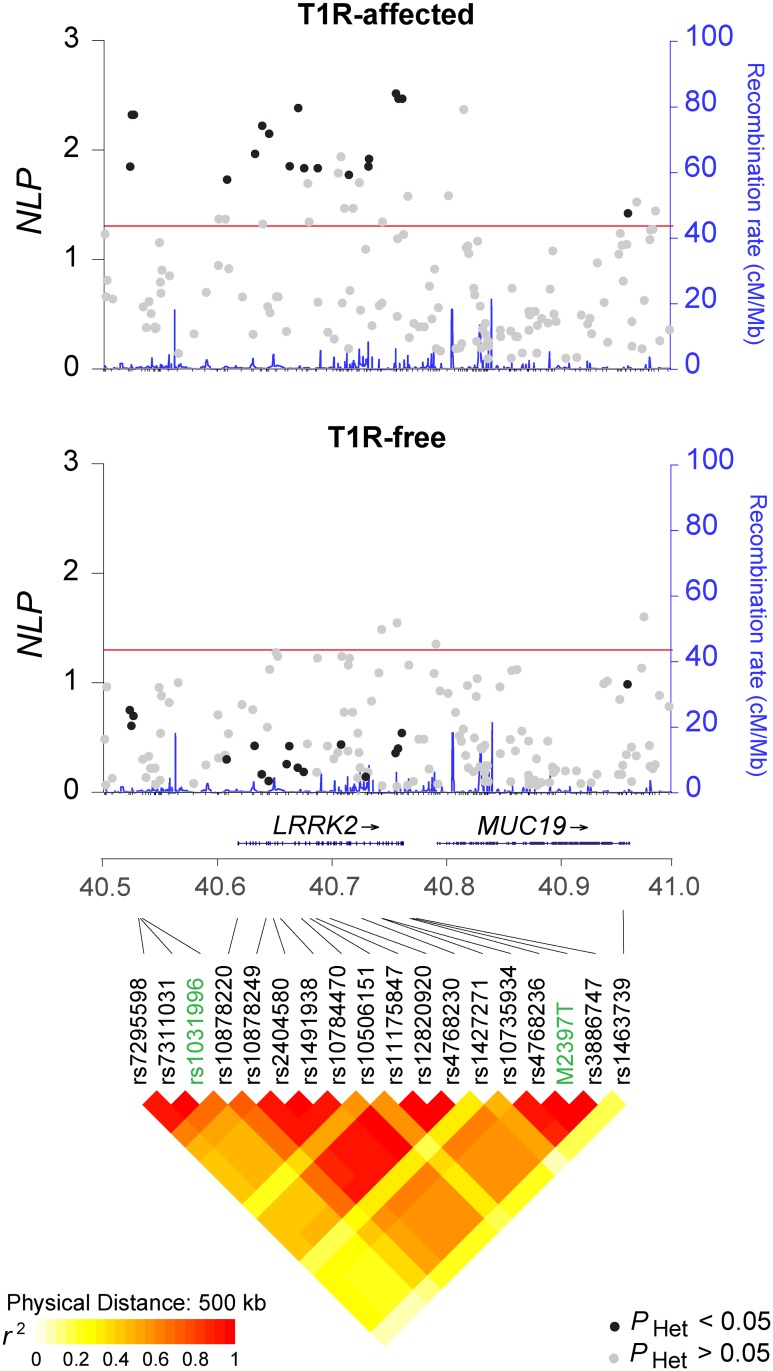
Association fine mapping of the *LRRK2* locus in the T1R-affected and T1R-free family subsets. Association results for 156 SNPs mapping to a 500 kb genomic interval encompassing the *LRRK2*/*MUC19* genes are shown at the top (T1R-affected) and at the center (T1R-free) of the graph. The SNPs are plotted according to their chromosomal position (GRCh37) on the x-axis against their negative log^10^ of association *P*-values (*NLP*) on the left y-axis. The blue line indicates the corresponding recombination rate in centimorgan per mega-base (cM/Mb) according to the 1000 genomes project (right y-axis). The red line symbolizes the 0.05 threshold for significant associations. SNPs that displayed significant evidence for heterogeneity of association with disease in the two family sets are indicated in black. At the bottom, the diamond plot represents the linkage disequilibrium pattern among the 18 SNPs preferential associated with T1R. The strength of pairwise linkage disequilibrium (*r*^2^) is indicated by color with yellow indicating weak LD and red strong LD.

To further evaluate the association of *LRRK2* with T1R, an additional 150 SNP were selected from the *LRRK2* gene region. Of all 156 SNPs evaluated for association in the T1R-affected subset, 34 showed *P* < 0.05 including the initial three SNPs associated with T1R (Fig 2). In the T1R-free family subset, the SNP rs7972711 situated near the 3’ end of the *MUC19* gene and rs10878434, rs7303525 and rs11564172 located near 3`end of *LRRK2* provided evidence of association (*P* < 0.05; Fig 2). None of the 34 SNPs associated with T1R showed evidence of association with leprosy in the T1R-free families (Fig 2). When formally tested for heterogeneity of association, 18 out of the 34 SNPs were preferentially associated with T1R (Fig 2 and Table 1). These 18 SNPs preferentially associated with T1R belong to two extended SNP bins (*r*^2^ > 0.5) and two single SNP bins (Table 1). Notably, although not nominally significant these 18 SNPs showed the opposite allelic enriched in T1R-free subset relative to the T1R-affected subset (Table 1). The bin tagged by the missense M2397T variant (rs3761863) situated in the WD40 domain of LRRK2 displayed the strongest preferential association with T1R (*P* = 0.003; odds ratio (OR) = 1.49; 95% confidence interval (CI) = 1.12–1.97 and *P*_Het_ = 0.008 for M2397 allele under an additive model) in the same direction as previously reported for CD (Table 2).

**Table 2:** Multivariate analysis of *LRRK2* variants with T1R

	SNP	rs1031996	M2397T	rs1427271	rs1463739
	*M*/*m*	C/T	M/T	G/A	A/G
	LD bin (*r*^2^ > 0.5)	Bin 1	Bin 2	Bin 3	Bin 4
T1R-affected	RA (*f*)	C (0.70)	M (0.51)	A (0.36)	A (0.74)
	*P*_uni_	0.005	0.003	0.01	0.04
	*P*_multi_	0.05	0.009	0.98	0.13
Reported associations	*RA* (*f*)^a^	C (0.47)	M (0.33)	A (0.14)	A (0.68)
	IBD				
	OR (95% CI)	1.03 (1.01–1.05)	1.08 (1.06–1.11)	1.10 (1.08–1.13)	1.02 (0.99–1.06)
	*P*	0.002	8.0E-15	3.2E-15	0.18
	CD				
	OR (95% CI)	1.04 (1.01–1.06)	1.12 (1.10–1.15)	1.16 (1.12–1.19)	1.00 (0.95–1.05)
	*P*	0.004	7.8E-21	3.5E-19	0.96
	UC				
	OR (95% CI)	1.03 (1.01–1.06)	1.03 (1.01–1.06)	1.03 (1.00–1.07)	1.03 (0.99–1.06)
	*P*	0.01	0.02	0.06	0.15

T1R, type-1 reaction; LRRK2, leucine-rich repeat kinase 2; M, major allele; m, minor allele; LD, linkage disequilibrium; RA, risk-allele; f, allele frequency; P uni from univariate analysis; P multi, P values for the multivariate analysis with the best SNP per bin. IBD, inflammatory bowel disease; CD Crohn’s disease; UC, ulcerative colitis; a Frequencies were based on the IBD sample. [22]

### Two independent associations signals with T1R in the *LRRK2* locus

To test if the *r*^2^ > 0.5 SNP bins were independently associated with T1R we performed a multivariate analysis including the most significantly associated SNP for each bin (Table 2). The multivariate analysis identified the missense M2397T variant as the main association with T1R (*P*_multi_ = 0.01; Table 2). However, a trend for independent association was observed for the SNP bin tagged by rs1031996 (*P*_multi_ = 0.05) and the single SNP bin rs1463739 (*P*_multi_ = 0.13; Table 2) while the association of T1R and rs1427271 disappeared.

We further investigated the combined effect of M2397T and rs1031996 by conducting a haplotype analysis. We found that the M2397 allele was mostly observed in the presence of the rs1031996 C allele (48% of the T1R-affected offspring) and this haplotype displayed a strong risk effect (Table 3). Importantly, the trend towards protection observed on the T2397 background was not modulated by rs1031996 alleles. Hence, the haplotype analysis confirmed that the main effect of the *LRRK2* gene on T1R susceptibility was driven by the M2397T variant. No haplotype association was observed when leprosy *per se* was considered as phenotype.

**Table 3:** Haplotype analysis of independent signal of association with T1R.

Haplotypes	(*f*)	Z	*P*
M2397T—rs1031996			
M—C	0.48	2.99	0.003
T—T	0.25	-1.74	0.08
T—C	0.23	-1.70	0.09
M—T	0.04	-0.54	0.59

*f*, allele frequency; *P* from univariate analysis

### Abrogation of genetically controlled differences in *LRRK2* expression by *M*. *leprae* sonicate stimulation

**Fig 3:**
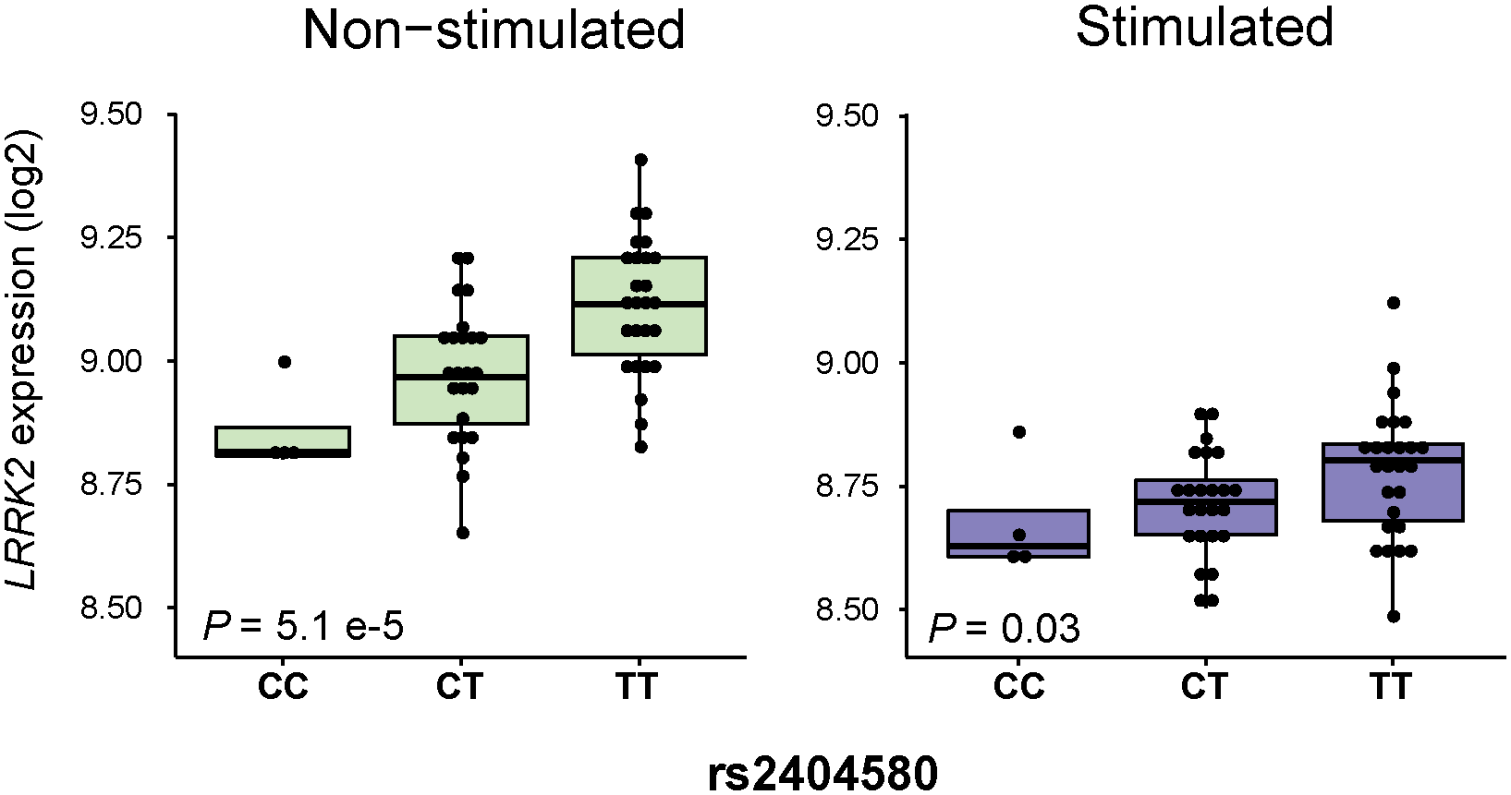
Host versus pathogen control of *LRRK2* expression levels. *LRRK2* expression levels for 53 unrelated subjects are indicated on the y-axis and stratified according to rs2404580 genotypes on the x-axis. The left panel represents baseline expression while the right panel indicates gene expression levels following stimulation with *M*. *leprae* antigen.

To investigate if SNP alleles associated with T1R were correlated with *LRRK2* transcriptional levels we performed an eQTL analysis. Of the 18 SNPs preferentially associated with T1R, nine variants belonging to the same SNP bin as the missense M2397T variant (*r*^2^ = 0.5) were eQTLs for *LRRK2* in non-stimulated whole blood of 53 individuals (Table 1, S1 Fig). When a tighter LD threshold (*r*^2^ = 0.8) was considered, the eQTL variants were separated from the bin tagged by M2397T. This observation suggested that the modest eQTL effect of M2397T was due to the linkage disequilibrium with the causal eQTL. The strongest eQTL effect was observed for SNP rs2404580 with the T1R-risk “T” allele being associated with higher *LRRK2* expression in unstimulated cells (*P* = 5.1E-05; Fig 3). Following stimulation with *M*. *leprae* sonicate, an abrogation of the eQTL effect was observed for all nine SNPs (Figs 3 and S1). Clinical subtypes of leprosy had no detectable impact on the eQTL effect of *LRRK2* genotypes.

## Discussion

**Fig 4:**
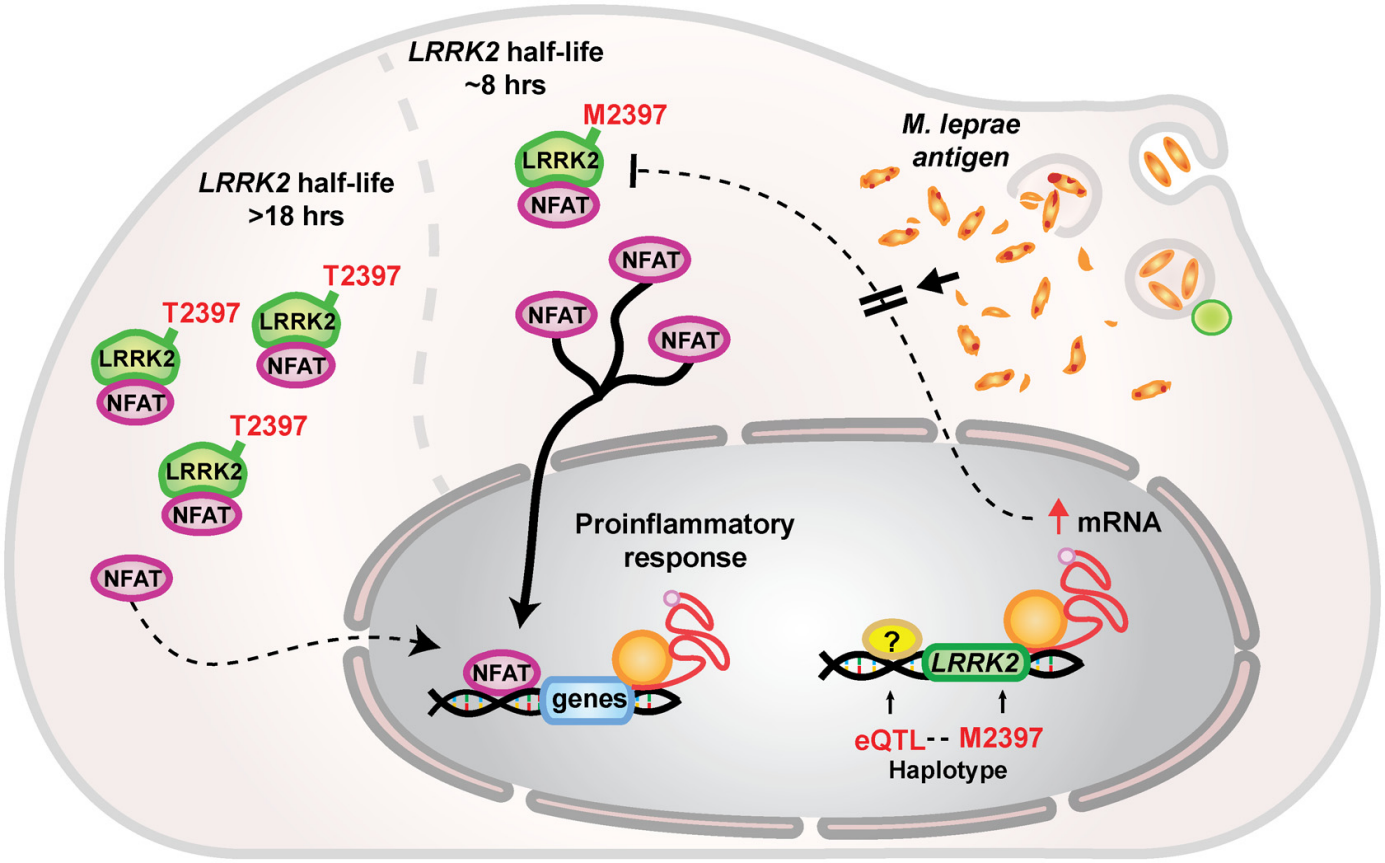
Proposed mechanism for LRRK2 in T1R. The LRRK2 M2397T amino acid substitution affects protein turnover. The methionine variant of LRRK2 displays a half-life of approximately 8 hours while the half-life of the threonine variant is 18 hours [34]. LRRK2 arrests the NFAT transcription factor in the cytoplasm through a complex mechanism mediated by Ca^2+^ [36]. This prevents NFAT to migrate to the nucleus and trigger the expression of pro-inflammatory cytokines [35]. The M2397 allele is in tight linkage disequilibrium with alleles of SNPs that promote an increase in *LRRK2* expression creating a compensatory mechanism to counterbalance the shorter LRRK2-M2397 half-life. This compensatory mechanism is abrogated in the presence of *M*. *leprae* antigen. Hence, the effect of the M2397T amino acid substitution is most pronounced in the presence of *M*. *leprae* antigen.

We identified an amino acid change M2397T in the WD40 domain of LRRK2 and a bin tagged by the variant the variant rs1031996 as being associated specifically with T1R. LRRK2 is a protein that exerts a diverse set of functions. LRRK2 mediates catalytic processes through its enzymatic ROC/COR domain; facilitates signal transduction through a MAPK domain and interacts with other proteins through three scaffold domains, an ankylin repeat (ANK), a leucine rich repeat (LRR) and a WD40 repeat domain [33]. Scaffold domains are also responsible for protein conformation and stability [33]. Although, LRRK2 displays multiple functions, the association of T1R with a variant in the WD40 domain of LRRK2 suggests that protein conformation and/or stability are key factors in T1R. Indeed, the M2397T variant in LRRK2 has previously been shown to impact on LRRK2 protein turnover [34]. The half-life of *LRRK2* with the T1R-risk allele M2397 had been estimated at approximately 8 hrs which is substantially shorter than the estimated 18 hrs half-life of the T2397 allele of LRRK2 (Fig 4) [34]. Cytoplasmic LRRK2 forms a complex that arrests nuclear factor of activated T-cells (NFAT) in the cytoplasm [34]. A consequence of LRRK2 deficit in the cytoplasm is the translocation of NFAT to the nucleus, which strongly induces the transcription of pro-inflammatory cytokines (Fig 4) [35, 36]. Thus, the association of the M2397 allele with T1R-risk is in agreement with an exacerbated pro-inflammatory response in T1R cases (Fig 4). While the M2397T variant is a strong candidate for functional impact in T1R, the association of the bin tagged by rs1031996 requires further investigations.

A set of eQTLs for *LRRK2* was observed in non-stimulated cell from leprosy patients. Due to LD the M2397 allele and the eQTL alleles that correlated with increased *LRRK2* expression were preferentially observed on the same haplotype. This suggests that the effect of M2397 on faster LRRK2 turn-over is mitigated by higher levels of *LRRK2* message. Interestingly, in the presence of mycobacterial antigen the compensatory effect of higher *LRRK2* message for the more rapid turnover of the M2397 protein is strongly abrogated. This implies that the genetic effect of the M2397 allele will be more pronounced in the presence of mycobacterial antigen while it may be largely abolished without such antigen exposure. While these *in-vitro* findings in whole blood will need to be validated employing purified cell types and *in-vivo* studies in leprosy lesion, the results obtained provide an example of how environmental stimuli can modulate germline encoded genetic risk factors (Figs 3 and 4).

*LRRK2* variants associated with T1R overlapped previous reported *LRRK2* associations with CD and PD [21, 22, 32]. In PD, rare coding variants in the enzymatic and kinase domains of *LRRK2* were shown to be causally linked to PD [33]. In addition, common *LRRK2* variants were shown in association with PD [32]. Two of these variants, rs1491932 and rs7970326, were observed in association with T1R and borderline evidence T1R specificity for the alleles opposite to PD. Common variants may tag rare variants with stronger effects. However, given our sample size we were unable to evaluate the role of rare variants in T1R. Of 18 SNPs preferentially associated with T1R, 17 were nominally associated with IBD with the same risk allele observed for T1R [22]. The only exception was rs1463739 a SNP located outside of *LRRK2* in the 3’ region of *MUC19* (Table 2 and Fig 2). When considering IBD subtypes, 14 T1R-risk variants were associated with risk of CD and 11 with risk of UC [22]. The M2397 allele is a risk factor for T1R, CD and UC, suggesting that a faster LRRK2 turnover leads to an increased pro-inflammatory response that is common to these diseases. T1R and CD may share susceptibility to mycobacterial species as common etiology [37, 38]. In PD, pathogen involvement is controversial although some studies suggested that *Helicobacter pylori* and prions might play a role in disease susceptibility [39–41]. Further studies will be needed to understand the precise role of LRRK2 in the pathogenesis of these three diseases.

## Supporting Information

S1 Fig A set of eQTL reported to the *LRRK2* gene. A to H present correlation of *LRRK2* expression with the genotypes of eight SNPs. These SNPs are significantly associated with T1R and belong to the same SNP bin (*r*2 > 0.5). *LRRK2* transcription levels are indicated on the y-axis for each of the three genotypes. Results for non-stimulated and *M*. *leprae* stimulated whole blood of 53 patients are presented in green and purple, respectively.
(TIF)

S1 Table Description of Vietnamese family-based sample employed in present study. (PDF)
