# Supplementary figures and images for: A Missense LRRK2 Variant Is a Risk Factor for Excessive Inflammatory Responses in Leprosy
Source: PLoS Negl Trop Dis. 2016 Feb 4;10(2):e0004412. doi: 10.1371/journal.pntd.0004412 (PMC4742274; doi:10.1371/journal.pntd.0004412)

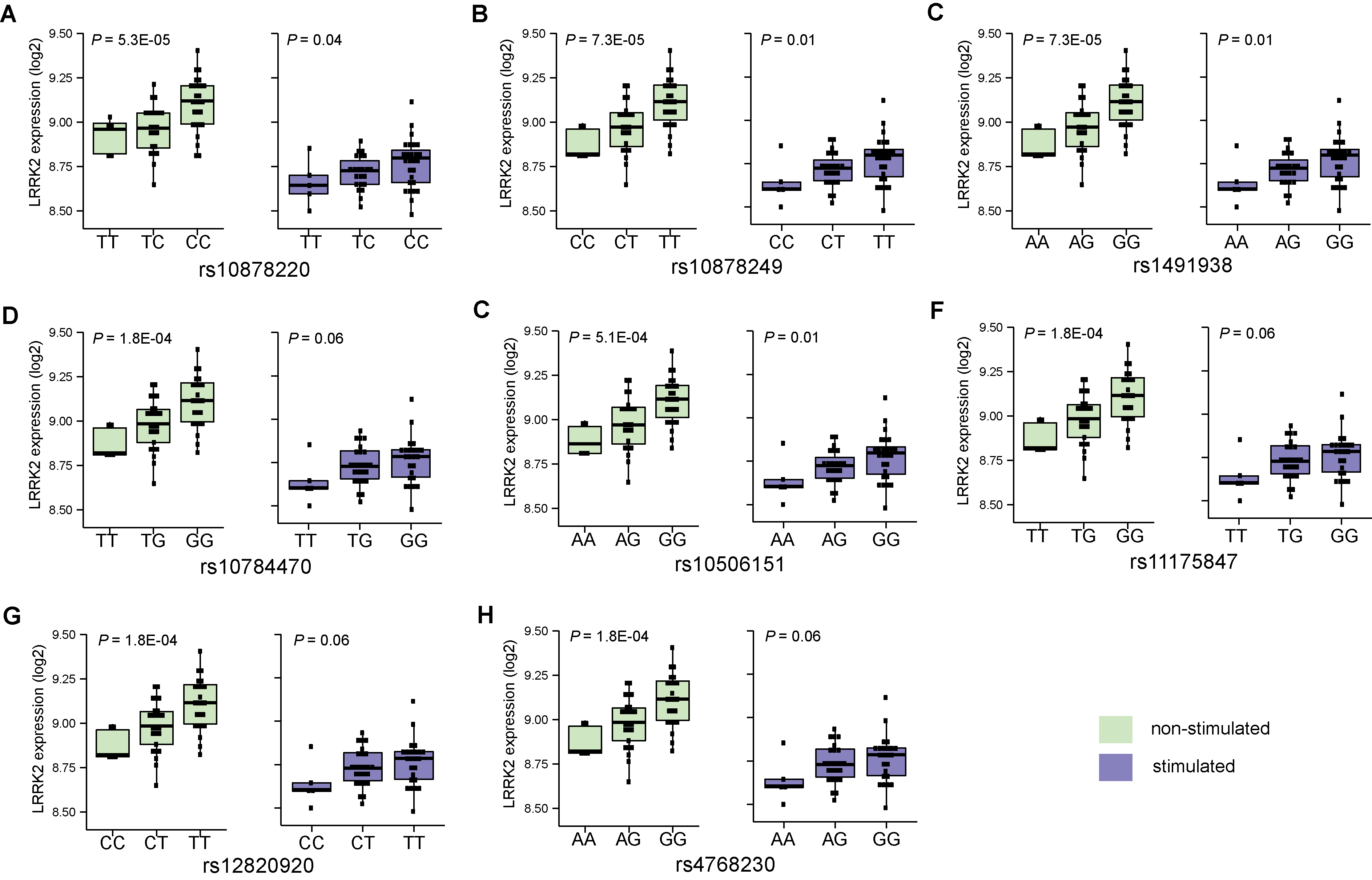

Supplement: S1 Fig — A to H present correlation of LRRK2 expression with the genotypes of eight SNPs. These SNPs are significantly associated with T1R and belong to the same SNP bin (r2 > 0.5). LRRK2 transcription levels are indicated on the y-axis for each of the three genotypes. Results for non-stimulated and M. leprae stimulated whole blood of 53 patients are presented in green and purple, respectively. (TIF) [file pntd.0004412.s001.tif]
