# Supplementary material for: A Missense LRRK2 Variant Is a Risk Factor for Excessive Inflammatory Responses in Leprosy
Source: PLoS Negl Trop Dis. 2016 Feb 4;10(2):e0004412. doi: 10.1371/journal.pntd.0004412 (PMC4742274; doi:10.1371/journal.pntd.0004412)
Supplement: S1 Table — (PDF) [file pntd.0004412.s002.pdf]

S1 Table 1. Vietnamese family-based sample.

	T1R-affected	T1R-free
	229	229
**Families**		
trios	213	189
2-sibs	8	20
**Gender**		
Male (%)	160 (69.9)	162 (70.1)
Female (%)	69 (30.1)	67 (29.9)
**Clinical subtype**		
TT	5	5
Borderline (BT / BB / BL)	215 (53 / 94 / 68)	215 (111 / 67 / 37)
LL	8	8
I	1	1
NP	–	–
**Age at leprosy onset**		
Mean (SD)	19.9 (7.6)	17.9 (7.1)

T1R, type-1 reaction; TT, tuberculoid pole; BT, borderline-tuberculoid; BB, borderline-borderline; BL, borderline-lepromatous; LL, lepromatous pole; I, Indeterminate; NP, neural pure; SD, standard deviation.
